# Host E3 ligase Hrd1 ubiquitinates and degrades H protein of canine distemper virus to inhibit viral replication

**DOI:** 10.1186/s13567-023-01163-z

**Published:** 2023-04-02

**Authors:** Wenjie Wang, Zhenwei Bi, Suquan Song

**Affiliations:** 1 Institute of Veterinary Medicine, Jiangsu Academy of Agricultural Sciences, Key Laboratory of Veterinary Biological Engineering and Technology, Ministry of Agriculture and Rural Affairs, National Center for Engineering Research of Veterinary Bio-Products, Nanjing, 210014 Jiangsu China; 2 MOE Joint International Research Laboratory of Animal Health and Food Safety, College of Veterinary Medicine, Nanjing Agricultural University, Nanjing, 210095 Jiangsu China; 3 GuoTai (Taizhou) Center of Technology Innovation for Veterinary Biologicals, Taizhou, 225300 Jiangsu China

**Keywords:** Canine distemper virus, Hrd1, H protein, ubiquitination, replication

## Abstract

Canine distemper (CD) is a highly contagious and an acutely febrile disease caused by canine distemper virus (CDV), which greatly threatens the dog and fur industry in many countries. Endoplasmic reticulum (ER)-associated degradation (ERAD) is a protein quality control system for the degradation of misfolded proteins in the ER. In this study, a proteomic approach was performed, and results found the E3 ubiquitin ligase 3-hydroxy-3-methylglutaryl reductase degradation protein 1 (Hrd1), which is involved in ERAD, as one of the CDV H-interacting proteins. The interaction of Hrd1 with CDV H protein was further identified by Co-IP assay and confocal microscopy. Hrd1 degraded the CDV H protein via the proteasome pathway dependent on its E3 ubiquitin ligase activity. Hrd1 catalyzed the K63-linked polyubiquitination of CDV H protein at lysine residue 115 (K115). Hrd1 also exhibited a significant inhibitory effect on CDV replication. Together, the data demonstrate that the E3 ligase Hrd1 mediates the ubiquitination of CDV H protein for degradation via the proteasome pathway and inhibits CDV replication. Thus, targeting Hrd1 may represent a novel prevention and control strategy for CDV infection.

**Supplementary Information:**

The online version contains supplementary material available at 10.1186/s13567-023-01163-z.

## Introduction

Canine distemper (CD) virus (CDV) was initially described as an infectious disease of domestic dogs, but it has increasingly become a known pathogen infecting many other species throughout the world. CDV has tropism to epithelial, lymphoid, and neurological cells, leading to systemic infection, including respiratory, digestive, urinary, lymphatic, cutaneous, skeletal, and central nervous system diseases [1]. CDV has caused serious economic losses to the fur animal and pet dog industry, and it is an important threat for wildlife conservation. Vaccination is the primary strategy to combat CDV infections, but the incidence of CD has increased worldwide in recent years [2]. Thus, CD is still a significant global epidemic infectious disease. In addition, CDV cross-species infections were reported in non-human primates, with high mortality rates [3, 4], which have raised several concerns of a potential zoonotic risk of CDV in humans.

The genome of CDV is a single-stranded negative RNA of 15 690 nucleotides, including six regions coding for nucleocapsid protein (N), phosphoprotein (P), large (L), matrix (M), hemagglutinin (H), and fusion protein (F), and untranslated regions (UTRs) [5, 6]. As an envelope protein, the H glycoprotein interacts with specific host cell receptors (SLAM, nectin-4, or others), which determine viral tropism. The H protein also triggers the fusion processes of F protein [7]. The CDV H protein is the main target for neutralizing antibody to prevent the infection of CDV [8]. Viruses induce multiple pathogenic effects due to the different interactions between the viral particle and the host [9]. The CDV H protein plays a crucial role in viral pathogenicity [10], but the CDV H protein-associated host factors, which are involved in viral replication and pathogenicity, remain predominantly unknown.

Endoplasmic reticulum (ER)-associated degradation (ERAD) is a protein quality control system for the degradation of misfolded proteins in the ER [11]. Most substrates of ERAD are polyubiquitinated by the E3 ubiquitin ligase. Hrd1 is anchored to the ER membrane [12]. The Hrd1 of 616 amino acids comprises an N-terminal signal peptide, six transmembrane domains, a RING-finger domain, and a C-terminal proline-rich domain. Hrd1 contains eight conserved cysteine and histidine residues in the core of its RING domain that is momentous for its E3 ubiquitin ligase activity, but it could be abolished by mutation of the cysteine residue at position 329 to serine (C329S) [13]. The transmembrane domain of Hrd1 is partially involved in the dislocation of the substrate from the ER to the cytosol and contributes to the stability of Hrd1 protein; the proline-rich domain is necessary for the interaction with substrate proteins [14]. Previous studies have identified Hrd1 as crucial for the replication of dengue virus (DENV) and Zika virus [15, 16]. The glycoprotein O (gO) of human cytomegalovirus (HCMV) could be degraded by Hrd1 in ERAD to inhibit viral infection [17]. Hrd1 was found to be anchored to the ER membrane [18], and the CDV H protein was reported to transfer to the ER for posttranslational modification [19, 20]. The roles of Hrd1 in the stability of CDV H protein and virus replication are unclear.

In this study, the host proteins associated with the H protein of CDV was screened using a proteomic approach. The interaction and co-location of the E3 ubiquitin ligase Hrd1 with CDV H protein were identified. The roles of Hrd1 in the stability of CDV H protein and viral replication were clarified. The results provide new insights into the role of Hrd1 in CDV infection and suggest a novel strategy for anti-CDV drug development.

## Materials and methods

### Cells, viruses, and viral titer assays

Vero and 293 T cells were cultured in Dulbecco modified Eagle medium (Gibco) supplemented with 10% fetal bovine serum (Gibco) and antibiotic–antimycotic solution (Gibco) at 37 °C in a 5% CO_2_ atmosphere. CDV 851 strain was available in our lab [21], and it was cultured in Vero cells with 10^5^ TCID_50_/mL. The viral titers in the culture supernatants of CDV 851-infected Vero cells were determined by the Reed–Muench method.

### Antibodies and reagents

The antibody information involved in the experiment was as follows: anti-Flag mouse monoclonal antibody (F1804, Sigma), anti-Ha mouse monoclonal antibody (BD-PM2095, Biodragon), anti-Hrd1 rabbit polyclonal antibody (A2065, Abclonal), anti-Myc mouse monoclonal antibody (ab206486, Abcam), anti-CDV H mouse monoclonal antibody 4C6 and anti-NP mouse monoclonal antibody G3N (homemade in our lab) [21], anti-GAPDH mouse monoclonal antibody (60004-1-Ig, Proteintech), HRP-conjugated goat anti-mouse IgG (BF03001, Biodragon), Alexa Fluor 488-conjugated AffiniPure Goat Anti-Mouse IgG (H + L) (AS076, Abclonal), and Alexa Fluor 594-conjugated AffiniPure Goat Anti-Rabbit IgG (H + L) (AS074, Abclonal). MG132 was purchased from MedChemExpress (USA). The reagent jetPRIME was purchased from Polyplus Transfection (France).

### Plasmid construction

The CDV 851 H protein was cloned into the mammalian expression vector pCAGGS-Flag for constructing pCA-Flag-H-WT. The K7R, K75R, K80R, K115R, K197R, K370R, and K455R of pCA-Flag-H-WT were mutated by PCR. The Hrd1 gene was amplified from 293 T cells by RT-PCR, and then cloned into the mammalian expression vector pCAGGS-Myc or pCDNA-3.1-Ha for constructing pCA-Myc-Hrd1 or pCDNA-3.1-Ha-Hrd1. PCR was used for C329S mutation of Hrd1. Wild-type Ub and mutated Ub (K48, K48R, K63, and K63R) were synthesized by the Tongyong Company (Anhui, China), and then cloned into the mammalian expression vector pCDNA3.1-Ha. All expression plasmids were verified by sequencing (TsingKe, China).

### Mass spectrometry

293 T cells were grown to 80–90% confluence in 6-well plates and transfected with the Flag-CDV H or vector. At 24 h post-transfection, the cells were rinsed three times with PBS, followed by incubation with ice-cold NP-40 lysis buffer (Beyotime) for over 1 h. The cell lysates (Flag-tagged proteins) were immunoprecipitated using anti-Flag antibody at 4 °C overnight. New protein A/G plus agarose was added for 4 h at 4 °C with gentle rotation. After being washed with NP-40 buffer, a small fraction of eluent was subjected to Western blot analysis. The rest of the eluted proteins were used for mass spectrometry by the GeneCreate Biological Engineering Company (Wuhan, China).

### Co-IP assay

293 T cells were transfected with Flag-CDV H and Myc-Hrd1 plasmids for 24 h, and the empty vector (Flag or Myc) was used as the control. After the cells were lysed, the cell lysates were centrifuged at 12 000 *g* for 10 min at 4 °C. The supernatants were collected and incubated with anti-Flag or anti-Myc antibody at 4 °C overnight and subsequently with protein A/G agarose beads for 4 h at 4 °C to rotate equably for mixing. Then, the immunoprecipitated beads were washed and eluted by NP-40 buffer as IP samples. Vero cells were infected with CDV 851 at an MOI of 0.1, and then lysed at 72 h post-infection with NP-40 buffer. After centrifuging, the supernatant was immunoprecipitated by mixing equably with anti-CDV H antibody overnight and subsequently with protein A/G agarose beads for 4 h at 4 °C. The immunoprecipitated beads were washed and eluted by NP-40 buffer. The input and IP samples were subjected to Western blot using corresponding antibodies.

### Detection of ubiquitination by IP

For the ubiquitination of CDV H, 293 T cells were transiently transfected with Flag-CDV H, Ha-Ub, and Myc-Hrd1 or its E3 ligase-dead mutant C329S for 24 h, and then the cells were lysed in NP-40 buffer. The lysates were spun at 12 000 *g* for 10 min, and the clarified supernatants were incubated with anti-Flag antibody for 12 h, followed by protein A/G plus agarose beads for 6 h at 4 °C. The beads were washed and eluted with NP-40 buffer, and then the ubiquitination of CDV H was evaluated by Western blot. The ubiquitination types and sites of CDV H were identified as above.

### RNA extraction and RT-qPCR

Total RNA was extracted using the RNAiso reagent (TaKaRa). Equal concentrations (500 ng) of the obtained RNA were used for cDNA synthesis using the Evo M-MLV Reverse Transcription kit (Accurate Biology) in accordance with the manufacturer’s instructions. The resulting cDNA were then amplified by real-time PCR through the SYBR Green Master (Yeasen) with specific primers as follows: CDV-N forward: 5′-ACAGATGGGTGAAACAGC-3′, CDV-N reverse: 5′-CTCCAGAGCAATGGGTAG-3′, GAPDH forward: 5′-GTGGTGCTAAGCGTGTTATCATC-3′, and GAPDH reverse: 5′-GGCAGCACCTCTGCCATC-3′. qPCR was performed on the StepOnePlus Real-Time PCR System, and the relative mRNA levels were calculated with the 2^−ΔΔCT^ method, including normalization to CT values of GAPDH.

### Western blot

Cell samples were lysed with NP-40 buffer and the cell lysates were centrifuged at 12 000 *g* for 15 min at 4 °C. Then, the lysates were subjected to SDS-PAGE and transferred to PVDF membranes. The membranes were blocked with 5% skim milk in PBS for 1 h at room temperature, followed by washing three times with PBST. The membranes were incubated with corresponding primary antibodies and HRP-conjugated secondary antibodies. Finally, ECL solution (Vazyme) was applied to bands to measure the protein expression levels with the Tanon-5200 Chemiluminescence Imager (Tanon Science and Technology Company).

### Confocal fluorescence microscopy

293 T cells were seeded in confocal dishes and cultured overnight before transfection with the Flag-CDV H or vector. After 24 h of transfection, the cells were washed with PBS and fixed with 4% paraformaldehyde (BL539A; Biosharp) for 30 min at room temperature. After being washed three times with PBS, the cells were permeabilized in 0.1% Triton X-100 for 15 min and blocked in PBS containing 1% BSA for 1 h at room temperature, followed by incubation with anti-Flag and anti-Hrd1 antibodies at 4 °C for 12 h. After being washed three times with PBS, the cells were incubated with goat anti-mouse IgG (HL)-Alexa Fluor 488 and goat anti-rabbit IgG (HL)-Alexa Fluor 594 for 1 h. Next, the cells were washed three times with PBS, and the nuclei were stained with DAPI (4′,6-diamidino-2-phenylindole) for 10 min. Finally, the samples were analyzed by confocal laser scanning microscopy (LSM880, Zeiss).

### Gene knockdown by siRNA

The sequence of siRNA targeting Hrd1 (forward: 5′-CCGCCAUGCUGCAGAUCAAtt-3′, reverse: 5′-UUGAUCUGCAGCAUGGCGGtt-3′) was synthesized in GenePharma (Shanghai, China) in accordance with the reference [22]. Vero and 293 T cells were seeded into 12-well plates, and transfection was performed using jetPRIME (Polyplus) according to the manufacturer’s protocol. The 293 T cells were transfected with siHrd1 or siNC (control siRNA) for 24 h. The cells were transfected with Flag-CDV H for 24 h again, and Western blot was used to measure the expression levels of the Hrd1 and CDV H protein. The Vero cells were transfected with siHrd1 or siNC for 24 h, and then infected with CDV 851 (MOI = 0.1). At 72 h post-infection, the cells were harvested, and the knockdown efficiency of Hrd1 and the expression of CDV N protein were assessed via Western blot.

### Statistical analysis

Data are presented as the mean ± standard deviation (SD). Statistical significance was calculated using SPSS and GraphPad Prism 8. ImageJ software was used to quantify the band intensities. Student *t*-test was used to analyze the statistical differences for comparisons between two groups. A *p* value < 0.05 was considered statistically significance. Asterisks in figures indicate statistical significance (*p < 0.05, **p < 0.01).

## Results

### Identification of CDV H protein-host protein interaction network

**Figure 1:**
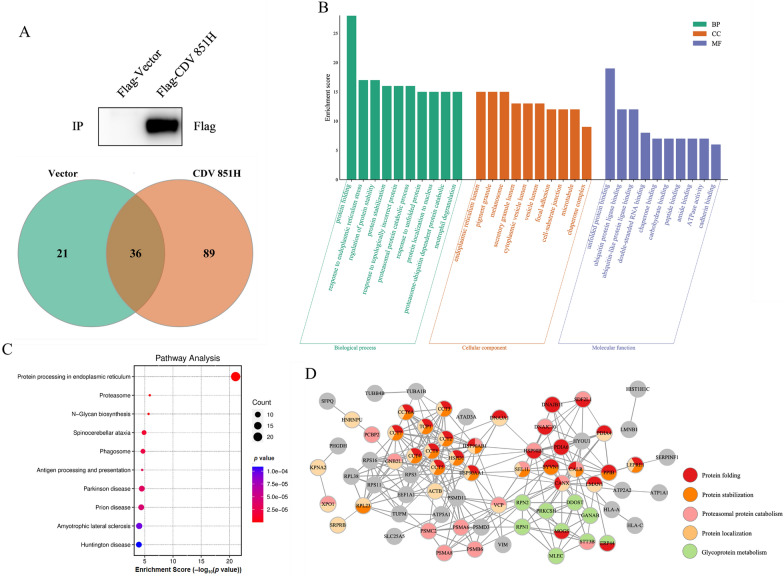
**Identification of CDV H protein–host protein interaction network.** **A** Identification of host proteins interacting with CDV H protein by mass spectrometry. 293 T cells were transfected with Flag vector, and Flag-CDV H for 24 h. Cell lysates were incubated with anti-Flag antibody overnight at 4 °C, and then protein A/G plus agarose was added for 4 h at 4 °C. The beads were washed and eluted with NP-40 buffer. A small fraction of eluents was subjected to Western blot with anti-Flag antibody, and a band of Flag-CDV H protein was visualized. The remaining eluents were analyzed by mass spectrum, and 89 specific proteins were identified to interact with CDV H protein in the Venn diagram. **B** GO analysis of CDV H-associated host proteins. Data show the top 10 GO terms enriched in molecular functions, biological processes, and cellular component functional groups. **C** KEGG analysis of CDV H-associated host proteins. **D** CDV H protein**–**host protein interaction map generated using Cytoscape software. Dark gray lines correspond to high-confidence PPI curated in the publicly available STRING database. Nodes with multiple colors indicate host proteins involved in multiple biological functions.

The underlying host proteins that may physically interact with the main structural H protein were identified by mass spectrometry providing information on the molecular mechanism of CDV infection. The host proteins associated with Flag-CDV H protein as bait were identified by mass spectrometry, and vector (Flag) was considered as the control. As shown in the Venn diagram of Figure 1A, a total of 89 host proteins were specific for binding to the CDV H protein. Gene oncology (GO) analysis shows that the 89 proteins were mainly enriched in protein folding, response to ER stress, regulation of protein stabilization and protein stability of biological processes: ER lumen, pigment granule, and melanosome of cellular component; and unfolded protein binding, ubiquitin protein ligase binding, and ubiquitin-like protein ligase binding of molecular function (Figure 1B). The Kyoto Encyclopedia of Genes and Genomes (KEGG) analysis shows that the proteins linked to CDV H protein were mainly involved in protein processes in the ER, proteasome and N-Glycan biosynthesis (Figure 1C). The CDV H protein-associated host proteins were submitted into the STRING database, and multiple proteins were selected to construct a protein–protein interaction (PPI) network by Cytoscape software (Figure 1D). The many known biological interactions were exhibited in the PPI map, such as the interaction between Hrd1 and SEL1L, which validate the results in this study. The results first suggest an interaction network between CDV H protein and host factors. The ER residence family members in the PPI network, such as CALR, Hsp90B1, PDIA4, VCP, SEL1L, and Hrd1, are implicated in protein folding, protein stabilization, and proteasome protein catabolism, all being involved in protein quality control in the ER. The best-defined function of the Hrd1 of these proteins is to ubiquitinate misfolded/unfolded proteins in the ERAD [23]. Consistently, in the following experiments, Hrd1 was found to be crucial for H protein stability and viral replication of CDV.

### Identification of the interaction between Hrd1 and CDV H protein

**Figure 2:**
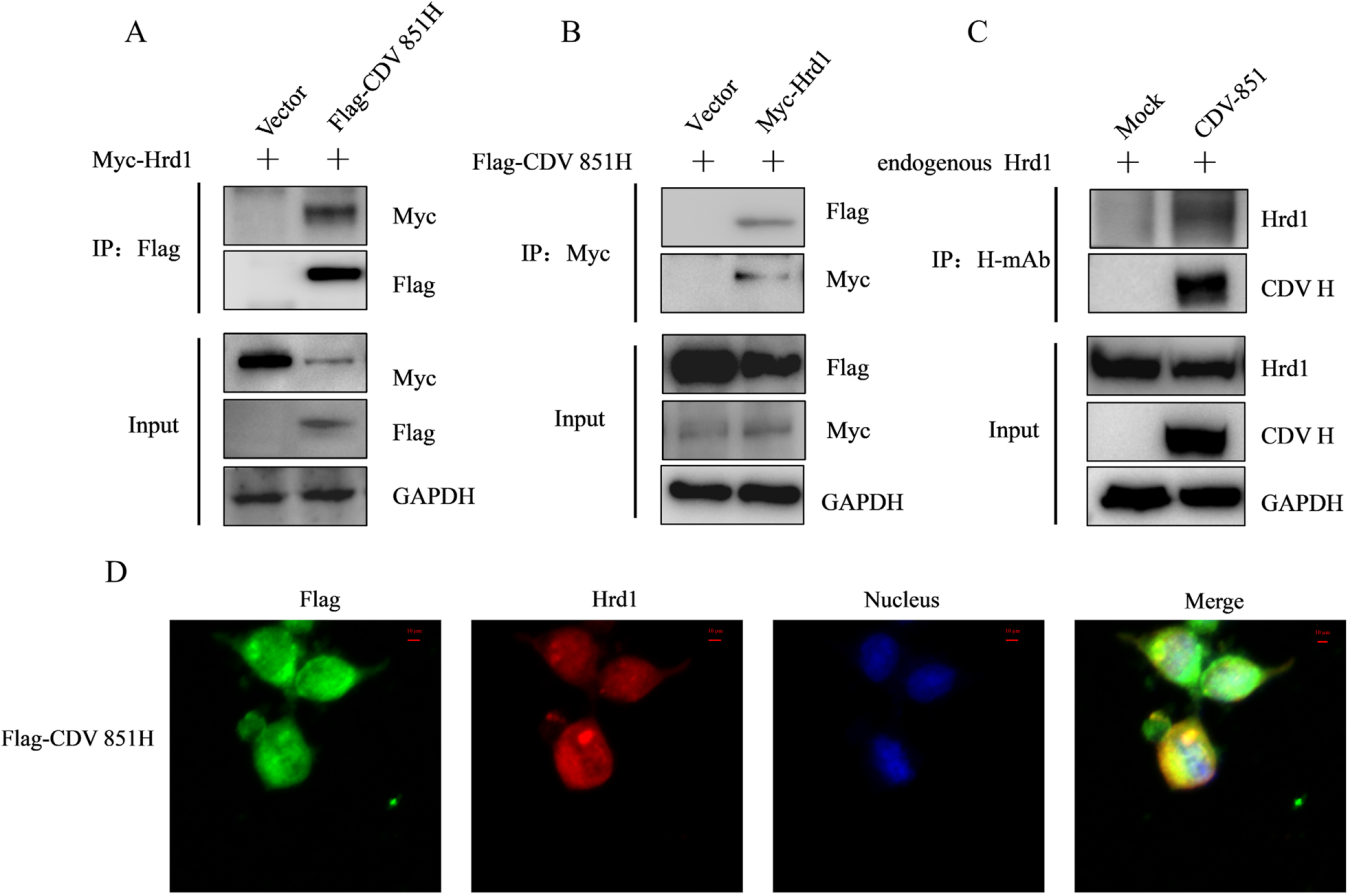
**Interaction and co-localization of Hrd1 with CDV H protein.** **A**,** B** Identification of the interaction between Hrd1 and CDV H protein by Co-IP. 293 T cells were co-transfected with Flag-CDV H and Myc-Hrd1 for 24 h, and Flag or Myc vector was used as the control. The transfected cells were lysed and immunoprecipitated with anti-Flag or anti-Myc antibody. The interactions were analyzed by Western blot with anti-Myc, anti-Flag and anti-GAPDH antibodies. **C** Identification of the interaction between Hrd1 and CDV H protein during viral infection by Co-IP. Vero cells were infected with CDV 851 (MOI = 0.1) for 72 h and then subjected to immunoprecipitation with anti-CDV H antibody. The interaction was analyzed by Western blot with anti-CDV H, anti-Hrd1 and anti-GAPDH antibodies. **D** Co-localization of Hrd1 with CDV H protein. 293 T cells were transfected with CDV H protein for 24 h. They were incubated with anti-Flag antibody and anti-Hrd1 antibody as primary antibodies and Alexa Fluor 488-conjugated AffiniPure Goat Anti-Mouse IgG (green) and Alexa Fluor 594-conjugated AffiniPure Goat Anti-Rabbit IgG (red) as secondary antibodies. Nuclei were stained with DAPI (blue). Co-localization between CDV H and Hrd1 was confirmed by confocal microscopy. Bars: 10 μm.

The E3 ubiquitin ligase Hrd1 was identified as one of the CDV H interacting partner candidates by mass spectrum. Co-IP assay was performed to further verify whether CDV H protein could interact with Hrd1. The Myc-Hrd1 protein was transfected alone or together with Flag-CDV H into 293 T cells for 24 h, and immunoprecipitation was performed with anti-Flag antibody. The results show that Myc-Hrd1 could be immunoprecipitated by the Flag-CDV H protein, whereas immunoprecipitation with Flag vector failed to couple with Myc-Hrd1 (Figure 2A). Next, the 293 T cells were transfected with Flag-CDV H protein alone or together with Myc-Hrd1 protein for 24 h, and the immunoprecipitates were bound with anti-Myc antibody. The results show that the Flag-CDV H protein could be immunoprecipitated by Myc-Hrd1 but not Myc vector (Figure 2B). Then, the interaction between endogenous Hrd1 and CDV H during viral infection was examined. The Vero cells were infected with CDV 851 strain for 72 h, and the cell lysates were immunoprecipitated by anti-CDV H antibody. The results show that endogenous Hrd1 protein could be only detected in CDV H protein-bound immunoprecipitates from CDV-infected Vero cells (Figure 2C). Thus, the endogenous Hrd1 and CDV H protein could interact with each other during viral infection. The co-localization of Hrd1 with CDV H protein was identified by confocal microscopy. The 293 T cells were transfected with Flag-CDV H or Flag-vector for 24 h, and then immunostained with anti-Flag and anti-Hrd1 antibodies. The inspection of 293 T cells transfected with Flag-CDV H, but not the empty vector, show yellow fluorescence (Figure 2D), revealing co-localization of CDV H protein with endogenous Hrd1 protein. Taken together, these results demonstrate that the host factor Hrd1 could interact with the CDV H protein.

### Hrd1 degrades CDV H protein via the proteasome pathway

**Figure 3:**
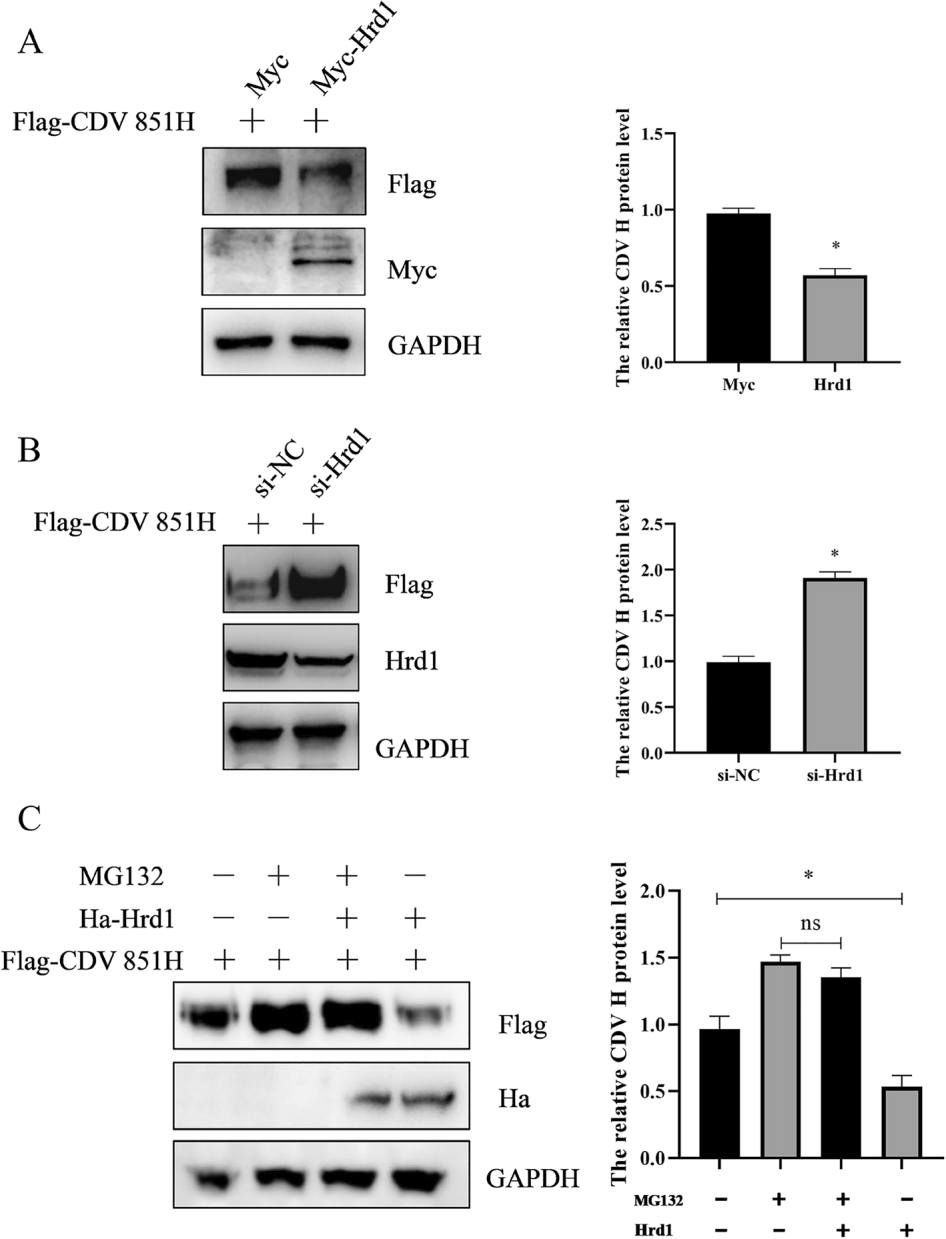
**Effect of Hrd1 on CDV H protein level.** **A** Overexpression of Hrd1-negatively regulated CDV H protein level. 293 T cells were transfected with Flag-CDV H alone or together with Myc-Hrd1 for 24 h, the protein levels were determined by Western blot using anti-Flag, anti-Myc, and anti-GAPDH antibodies. The level of CDV H protein was quantified by determining band intensities, which were then normalized to the level of GAPDH. **B** Increased CDV H protein level by knockdown of Hrd1. 293 T cells were transfected with siRNA for Hrd1 for 24 h and then transfected with Flag-CDV H for an additional 24 h. The indicated proteins were visualized by Western blot using anti-Flag, anti-Hrd1, and anti-GAPDH antibodies. The level of CDV H protein was quantified by determining band intensities, which were then normalized to the level of GAPDH. **C** Hrd1 degradation of CDV H protein through proteasome pathway. 293 T cells were transfected with Flag-CDV H alone or together with Ha-Hrd1 for 24 h and then treated with or without MG132 for 6 h. Protein levels were assessed by Western blot using anti-Flag, anti-Ha and anti-GAPDH antibodies. The level of CDV H protein was quantified by determining band intensities and subsequent normalization to the level of GAPDH. All results are shown as means ± SD from at least three separate sample preparations. * *p* < 0.05, ** *p* < 0.01.

The E3 ubiquitin ligase Hrd1 catalyzes the ubiquitination of substrate proteins for degradation in ERAD [24]. Considering that Hrd1 interacted with CDV H protein, the effect of Hrd1 on the degradation of CDV H protein was tested. 293 T cells were transfected with Myc-Hrd1 or vector together with Flag-CDV H for 24 h, and then the CDV H protein level was examined by Western blot. The results show that overexpression of Hrd1 reduced the protein level of CDV H protein (Figure 3A). An RNA interference (RNAi) strategy was employed to knock down host factor Hrd1 to further verify the effect of Hrd1 on the degradation of the CDV H protein. The 293 T cells were transfected with siRNA for Hrd1 or si-NC for 24 h, and then transfected with CDV H protein for an additional 24 h. Western blot was used to detect the expression of CDV H protein and confirm the silencing efficacy of Hrd1-targeted siRNA. As shown in Figure 3B, the CDV H protein level could be restored when the Hrd1 protein level was knocked down, further suggesting that Hrd1 mediated the degradation of CDV H protein. A study showed that Hrd1 targets its interacting proteins for proteasome-mediated degradation [18]. The cells were transfected with CDV Flag-H alone or together with Myc-Hrd1 for 24 h and then treated with or without proteasome inhibitor MG132 for 6 h to investigate whether Hrd1 regulates the degradation of CDV H protein via the proteasome pathway. The results show that Hrd1-mediated degradation of CDV H protein was blocked in the presence of MG132 (Figure 3C), demonstrating that CDV H protein was degraded by Hrd1 through the proteasome pathway.

### E3 ubiquitin ligase activity is essential for Hrd1-mediated CDV H protein degradation

**Figure 4:**
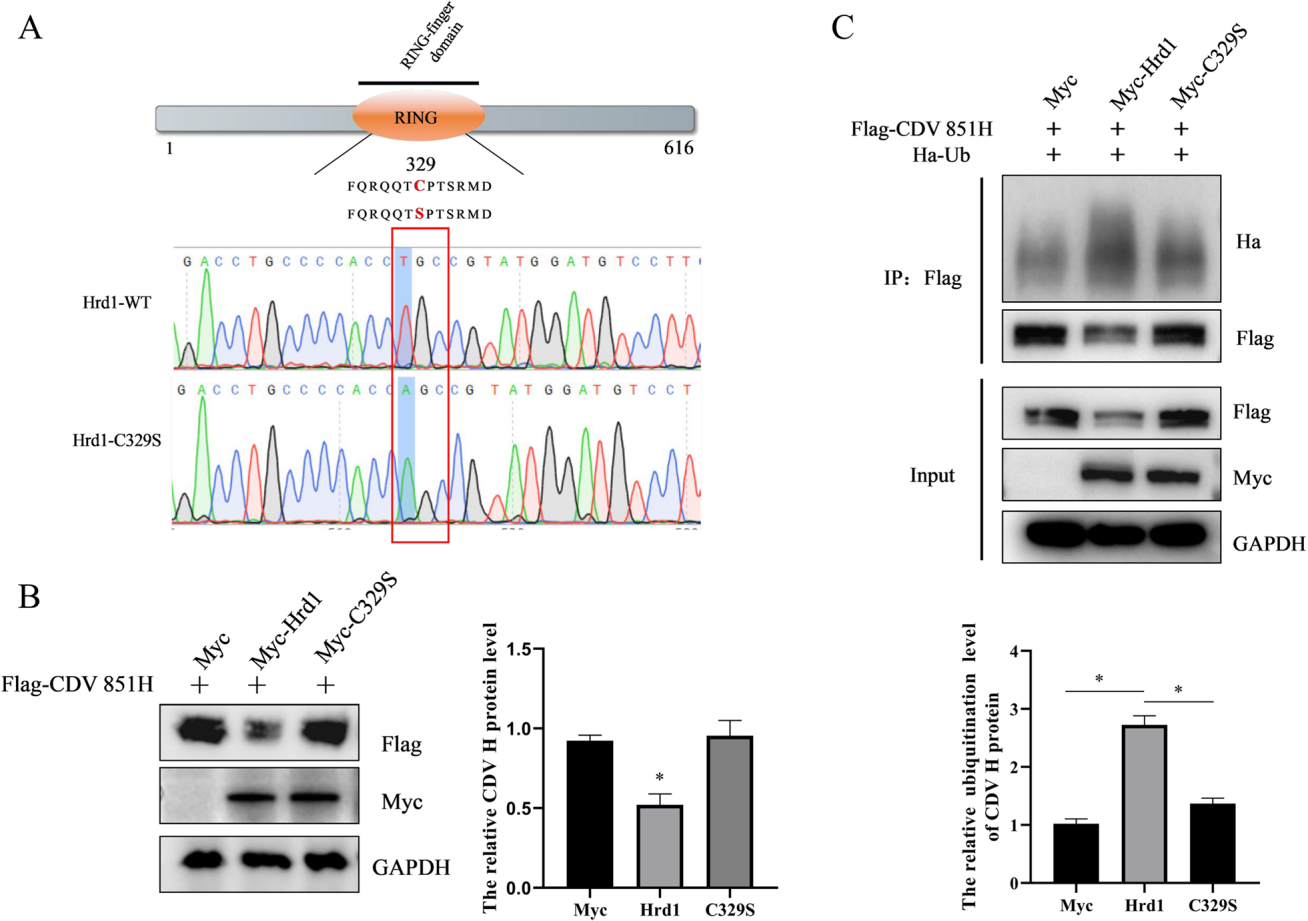
**Critical role of E3 ligase activity on Hrd1-mediated CDV H protein degradation.** **A** Schematic of the RING domain of Hrd1. The red C329S mutation in RING domain could abolish the E3 ligase activity of Hrd1. The partial DNA sequence that contains normal WT and mutant Hrd1 were aligned and the conserved cysteine residue in position 329 of the RING domain was replaced by a serine residue. **B** Hrd1 with C329S mutation did not degrade CDV H. 293 T cells were transfected with Flag-CDV H alone or together with Myc-Hrd1 or C329S mutant for 24 h, and proteins were analyzed by Western blot using anti-Flag, anti-Myc and anti-GAPDH antibodies. The level of CDV H protein was quantified by determining band intensities, which were then normalized to the level of GAPDH. Data are means ± SD (*p < 0.05). **C** Hrd1 with C329S mutation did not modify ubiquitination of CDV H protein. Ha-Ub and Flag-CDV H were co-transfected into 293 T cells together with Myc-Hrd1 or Myc-C329S mutant for 24 h. The cells were lysed and immunoprecipitated with anti-Flag antibody. The input samples and co-precipitated proteins were analyzed by Western blot with anti-Ha, anti-Flag, anti-Myc and anti-GAPDH antibodies. The ubiquitination level of CDV H protein was quantified by determining band intensities, which were then normalized to immunoprecipitated CDV H protein. Data are means ± SD (*p < 0.05).

The E3 ubiquitin ligase Hrd1 could catalyze the addition of ubiquitin molecules to lysine residues in substrate proteins to promote their degradation [18]. Here, Hrd1 was identified to promote the degradation of CDV H protein. Next, whether the E3 ligase activity of Hrd1 is required for its ability to degrade CDV H protein was assessed. Since previous reports indicate that the mutation (C329S) that occurs at position 329 of Hrd1 abolishes its E3 ligase activity [25], the C329S mutation was performed in the Myc-Hrd1 plasmid (Figure 4A). The CDV H protein level was detected after co-transfection with CDV H protein and the wild-type Hrd1/C329S mutant for 24 h, and then the cells were lysed to examine the expression of CDV H protein by Western blot. Compared with wild-type Hrd1, the catalytic inactive mutant Hrd1 (C329S) failed to degrade the H protein of CDV 851 (Figure 4B). In addition, whether the inactivation of E3 ligase activity of Hrd1 could affect the ubiquitination of CDV H was determined. 293 T cells were co-transfected with Flag-CDV H, Myc-Hrd1/C329S mutant, and Ha-Ub for 24 h, and then an immunoprecipitation assay was performed using anti-Flag antibody. Increased ubiquitination of CDV H was observed in the presence of Hrd1, whereas CDV H ubiquitination apparently decreased when the E3 ubiquitin ligase activity of Hrd1 was inactive (Figure 4C). These results indicate that the E3 ligase Hrd1 catalytic activity is required for Hrd1-mediated degradation of the CDV H protein.

### Hrd1-mediated K63-linked polyubiquitination at position K115 of CDV H protein

**Figure 5:**
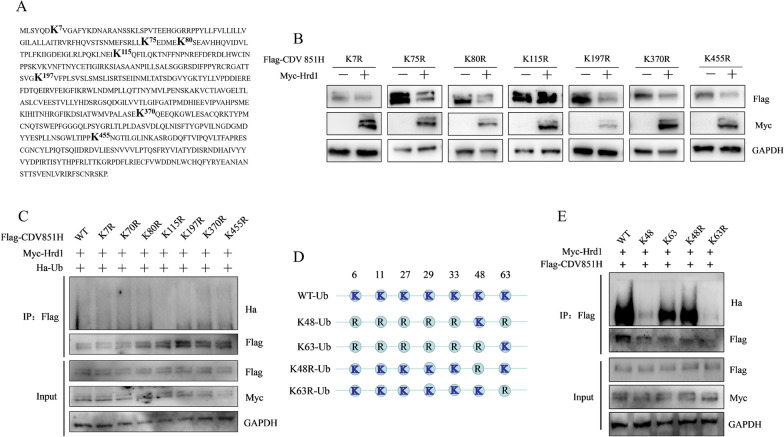
**Hrd1-mediated K63-linked polyubiquitination of CDV H at position K115.** **A** Schematic of amino-acid sequence of CDV H. Potential ubiquitin lysine residues are displayed in boldface. **B** CDV H protein with K115R mutation was not degraded by Hrd1. 293 T cells were transfected with the indicated plasmids for 24 h, and then cell lysates were analyzed by Western blot using anti-Flag, anti-Myc and anti-GAPDH antibodies **C** K115R mutant of CDV H eliminated Hrd1-mediated ubiquitination. 293 T cells were co-transfected with the indicated plasmids for 24 h, and then the cell lysates were subjected to ubiquitination assays and Western blot with anti-Flag, anti-Ha, anti-Myc and anti-GAPDH antibodies. **D** Schematic of ubiquitin K48 and K63 mutants. **E** The ubiquitin of CDV H by Hrd1 was K63-linked. 293 T cells were co-transfected with the indicated plasmids for 24 h. The cell lysates were subjected to ubiquitination assays and Western blot with anti-Flag, anti-Ha, anti-Myc and anti-GAPDH antibodies.

The next important question was which lysine residues on CDV H protein are ubiquitinated by Hrd1. Thus, the site of Hrd1-mediated ubiquitylation within the CDV H protein was identified. Bioinformatics methods were employed to predict the possible ubiquitination sites (K7, K75, K80, K115, K197, K370, and K455) of CDV H (Figure 5A). H protein mutants bearing individual Lys-to-Arg substitutions were generated at these predicted sites to accurately determine the CDV H protein ubiquitination sites. 293 T cells were transfected with Myc-Hrd1 and mutated CDV H for 24 h, and then the expression of CDV H protein was tested using Western blot. The results show that mutation of K115R inhibited H protein degradation in 293 T cells expressing Hrd1, whereas the other mutants could still be obviously degraded by Hrd1 (Figure 5B). Therefore, K115, not the other lysine residues in the CDV H protein, was determined as the main acceptor of degradation catalyzed by Hrd1. Then, conservation analysis of K115 in the H proteins of 412 CDV strains of different genotypes, which are listed in Additional file 1 was conducted. The results show that K115 is highly conserved among CDV strains of different genotypes, and only one strain Wgswh02 (KX159296) of America-1 genotype had the K115R mutation (Additional file 2). Next, whether the K115 of CDV H protein is the ubiquitination site was investigated by a ubiquitination assay. As expected, compared with wild-type H protein, the ubiquitylation level decreased in the K115R mutant but not in the other mutants, suggesting that the CDV H protein evaded the ubiquitination by Hrd1 by changing K115 to Arg (Figure 5C). Taken together, the K115 of CDV H protein is the ubiquitylation site modified by Hrd1 for degradation. Flag-CDV H and Myc-Hrd1 were transfected into 293 T cells together with the WT-Ub or mutant ubiquitins (K48-Ub, K63-Ub, K48R-Ub, and K63R-Ub) (Figure 5D) to determine which type of polyubiquitin linkage to CDV H is catalyzed by Hrd1. The results of co-immunoprecipitation and Western blot show that K63 and K48R mutations, each of which contained K63 lysine available for polyubiquitination, promoted polyubiquitination of the CDV H protein as efficiently as the wild-type ubiquitin, whereas K48 and K63R mutations, each of which contained K63 lysine mutated to Arg, reduced the polyubiquitination of CDV H protein compared with the wild-type ubiquitin (Figure 5E). A lighter band in the K48 and K63R groups was also observed, indicating that Hrd1 also mediated the K48-link polyubiquitination of CDV H protein, but it was not the major type of polyubiquitin. These findings suggest that Hrd1 mediates the K63-linked polyubiquitination of CDV H protein. Collectively, the data demonstrate that Hrd1 mediates CDV H protein degradation by K63-linked polyubiquitination on lysine at position 115 of the H protein.

### Effect of Hrd1 on CDV replication

**Figure 6:**
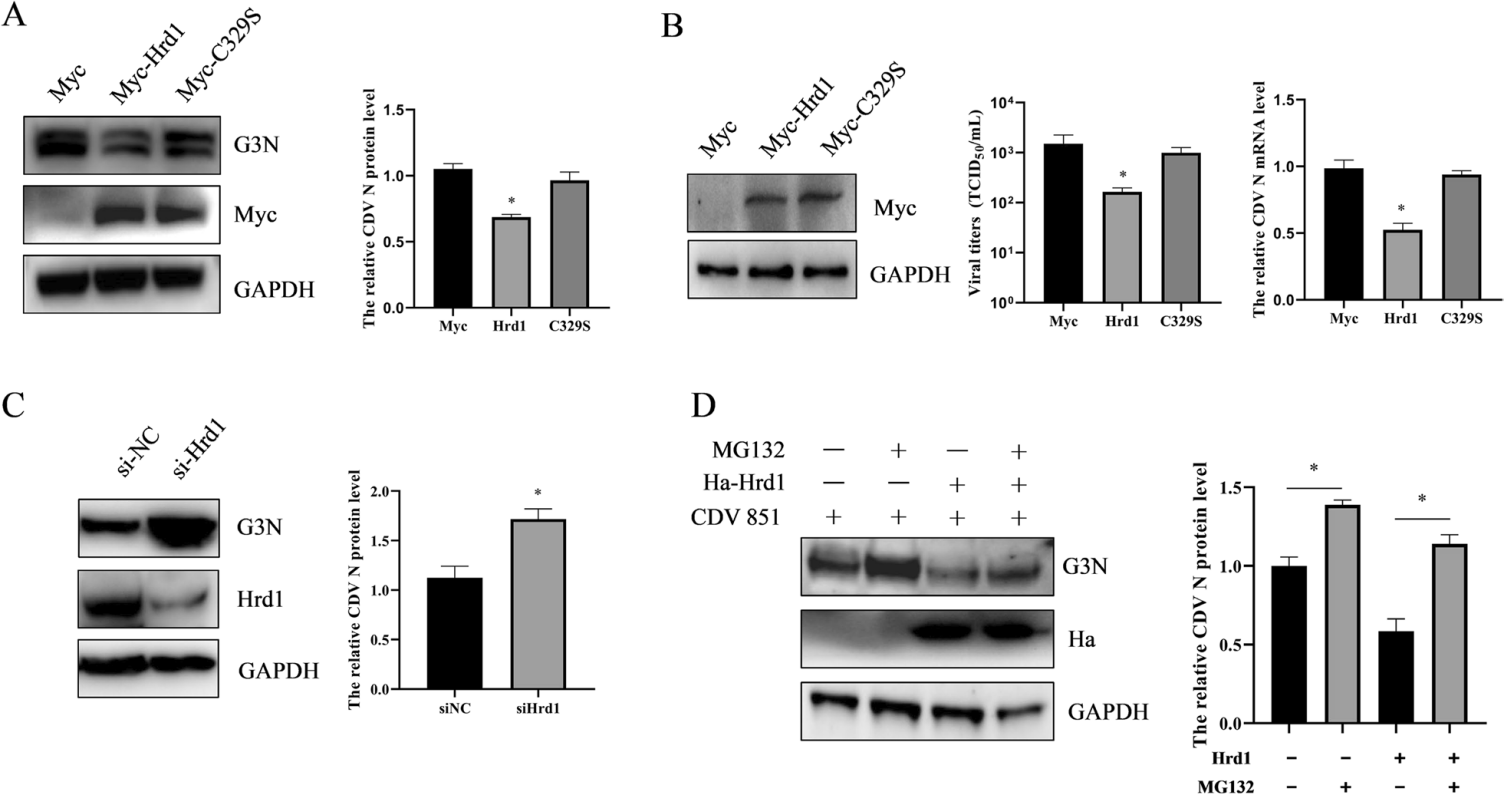
**Hrd1 inhibition of viral replication via its E3 ligase activity.** **A** Overexpression of Hrd1 inhibited the CDV N protein level during viral infection. Vero cells were transfected with Myc-Hrd1 or Myc-C329S mutant for 24 h, and then the transfected cells were infected with CDV 851 (MOI = 0.1) for an additional 72 h. The protein levels of cell lysates were determined by Western blot using anti-CDV N, anti-Myc, and anti-GAPDH antibodies. ImageJ was used to quantify the ratio of CDV N to GAPDH. **B** Overexpression of Hrd1 inhibited the N mRNA level and viral titers during viral infection. Vero cells were transfected with Myc-Hrd1 or Myc-C329S mutant for 24 h and then infected with CDV 851 for an additional 72 h. RT-qPCR was used for examination of mRNA levels of CDV N and GAPDH. The viral titer of culture supernatants were determined by the Reed–Muench method. **C** Knockdown of Hrd1 increased N protein level during viral infection. Vero cells were transfected with siRNA for 24 h and then infected with CDV 851 for an additional 72 h. Protein levels were determined by Western blot using anti-CDV N, anti-Hrd1, and anti-GAPDH antibodies. ImageJ was used to quantify the ratio of CDV N to GAPDH. **D** Blockage of the proteasome pathway restored the inhibition of viral replication by Hrd1. Vero cells were transfected with Ha-Hrd1 for 24 h and then infected with CDV 851 (MOI = 0.1) for 72 h in the presence of MG132 (20 μM). The lysates of harvested cells were subjected to Western blot analysis using anti-CDV N, anti-Ha and anti-GAPDH antibodies. Image J was used to quantify the ratio of CDV H to GAPDH. All results are shown as means ± SD from at least three separate sample preparations. *p < 0.05, **p < 0.01.

Whether Hrd1 affected the replication of CDV was further explored. Vero cells were transfected with Myc-Hrd1 or Myc-C329S-Hrd1 for 24 h and then infected with CDV 851 (MOI = 0.1). After infection for 72 h, the cells were harvested, and the proteins of Hrd1, CDV N, and GAPDH were tested by Western blot. The results show that overexpression of Hrd1 protein reduces the N protein level. When the E3 ligase activity of Hrd1 was inactivated by mutating C329S, the N protein was not obviously decreased during viral infection (Figure 6A). Comparatively, Hrd1 did not mediate the degradation of CDV N protein in a direct manner by co-transfecting the two plasmids of CDV N protein and Hrd1 in 293 T cells (Additional file 3). RT-qPCR was used to determine the transcript level of N gene of CDV in Vero cells expressing Hrd1. The transcript level of N shows a significant downregulation at 72 h with infected CDV 851 (MOI = 0.1) when Hrd1 was overexpressed, whereas the transcript levels did not significantly decrease in Vero cells expressing mutant Hrd1 protein with inactive E3 ligase activity (Figure 6B), consistent with the N protein level. In addition, the viral titer of the supernatant samples was determined by the Reed–Muench mothed. Similarly, the results show that overexpression of Hrd1, but not mutant Hrd1 (C329S), decreased the viral titer (Figure 6B). These results suggest that Hrd1 inhibited the CDV replication depending on its E3 ligase activity. Next, an RNA interference (RNAi) strategy was employed to knock down host factor Hrd1 in Vero cells. The Vero cells were transfected with Hrd1-targeted siRNA or siRNA control for 24 h, followed by virus infection at an MOI of 0.1 for an additional 72 h. The silencing efficacy of Hrd1-targeted siRNA in Vero cells was confirmed by Western blot. The Vero cells transfected with the indicated siRNA resulted in the efficient knockdown of Hrd1 at 96 h post-transfection. Meanwhile, Hrd1 knockdown mediated by siRNA significantly increased the N expression of CDV (Figure 6C). Considering that the E3 ligase activity was required for Hrd1-mediated CDV H protein degradation via the proteasome pathway, the assay that CDV infected the Vero cells expressing Hrd1 for 24 h in the presence of MG132 was performed. The results show that the viral N protein was reduced in the absence of MG132, but this could be restored by adding MG132 (Figure 6D). Overall, these results indicate that inhibition of CDV replication by Hrd1 depends on the E3 ligase activity of Hrd1 and the proteasome pathway.

## Discussion

The complex interactions between host and viral proteins play important roles in viral infection by different mechanisms [26]. A large number of host cellular proteins interacting with various viruses have been identified by Co-IP assay and mass spectrum, and the roles of the interactions in viral replication and pathogenicity have been explored [27]. However, the identity and function of the host cellular factors involved in CDV replication remain largely unknown. The CDV H protein is involved in receptor binding, mediation of membrane fusion, virus budding, and viral assembly. Thus, the stability of this protein is required for viral replication. In this study, a proteomic approach about CDV H protein-associated host cellular factors was performed to clarify which proteins participate in the stability of the CDV H protein. The mass spectral analysis demonstrates that the host factors that interacted with CDV H protein mainly include ER-related proteins. In fact, the CDV H protein was reported to transfer into the endoplasmic reticulum for glycosylation and modification. The interacting proteins are involved in biological effects such as protein folding, response to ER stress, protein stabilization, and ubiquitin protein ligase binding. ERAD plays a vital role in maintaining cellular homeostasis by recognizing terminally and misfolded polypeptides and removing them to the cytosol for proteasomal degradation. Furthermore, the interacting protein E3 ligase Hrd1, which is involved in ERAD, degraded the CDV H protein via the proteasome pathway. Thus, the H protein becomes the primary target for ERAD for degradation.

A growing body of evidence shows that viral proteins are ubiquitinated by recruiting host ubiquitin E3 ligases [28], which play key roles in viral replication. An important mechanism is the E3 ligase ubiquitinated viral protein for degradation. For example, the E3 ligase MARCH8 catalyzed ubiquitination of IAV M2 protein leading to M2 degradation for restricting IAV infection and pathogenesis [29]. TRIM69 ubiquitinated DENV NS3 to inhibit replication [30]. The ER resident E3 ubiquitin ligase Hrd1 is a key ERAD factor that directly catalyzes ubiquitin conjugation onto substrate proteins for degradation [17, 31, 32]. Previous studies reported CDV H protein finishing and processing in the ER [19, 20]. In the present study, the CDV H protein was found to interact and co-localize with Hrd1 by co-immunoprecipitation assays and confocal microscopy. Hrd1 promoted CDV H ubiquitination for its degradation via 26S proteasome pathway through E3 ligase activity. The ER-located RING-type E3 ligase Hrd1 was also found to be critical for the stability of the H protein. Next, the regulatory role of Hrd1 on CDV replication and propagation was explored. Hrd1 inhibited the replication of CDV, but it could be restored by proteasome inhibitor MG132 treatments. Hrd1 may negatively regulate CDV H protein stability and thus be responsible for the inhibition of CDV replication.

CDV H protein is considered as the most genetically variable gene, with up to 11% nucleotide divergence among CDV strains. Variant amino acid sites are available on the H protein of virulent and attenuated strains of CDV, and they affect the virulence of CDV strain and cross-species transmission [33, 34]. In the present study, the potential ubiquitination sites K7, K75, K80, K115, K197, K370, and K455 of CDV H protein was predicted through bioinformatics analysis, and the K115 site was confirmed as a specific site for CDV H protein ubiquitination mediated by Hrd1 through ubiquitination assay. Sequence analysis of different CDV strains shows that the K115 site is a highly conserved locus among the different strains. This finding suggests that almost all CDV H protein strains may be targeted by Hrd1. The recombinant A/Puerto Rico/8/34 (PR8) H1N1 IAV carrying the K78R M2 protein becomes resistant to MARCH8 and exhibits greater virulence in mice [29]. A notable detail indicating that CDV strains with K115R mutation may show strong virulence and pathogenicity. In addition, mutation of the 115 locus of CDV vaccine may increase the H protein levels and viral replication by antagonizing the viral protein degradation and antiviral effect of Hrd1, thus improving the immune effect of the CDV vaccine.

Ubiquitin is composed of 72 amino acid residues, including seven lysine residues; each lysine residue and N-terminal Met residues could form single or mixed polyubiquitin chain linkages [35]. Different linkage-specific ubiquitin chain modifications play different roles in various cellular signaling pathways, and they cross one another to form a complex regulatory network [36]. The most widely studied is the K48-linked ubiquitin chains, which mainly guide proteins to 26S proteasome for degradation. K63-linkage specific polyubiquitination regulates numerous functions of target proteins such as PPI intracellular localization, DNA repair, endocytosis, and transport. An increasing number of studies show the substrates including some viral proteins were modified by E3 ligase for K48- or K63-linked ubiquitination for degradation [37–42]. In the present study, Hrd1 degraded the CDV H protein. Thus, K48- or K63-linked ubiquitination was used to identify the specific ubiquitin chain linking to the CDV H protein by Hrd1. The data suggest that K63-linked chains are involved in Hrd1-mediated CDV H degradation, further verifying the function of K63-linked chains to mediate protein degradation. Ubiquitin ligases with the same structure often have similar selection of ubiquitin chains [43]. Hrd1 is considered as a member of the E3 ligase family containing RING structure [13], and some members of the RING-type E3 ligase, such as RNF168, RNF8, and RNF152, have been shown to mediate K63 ubiquitin chain modification of the substrate [44–46].

**Figure 7:**
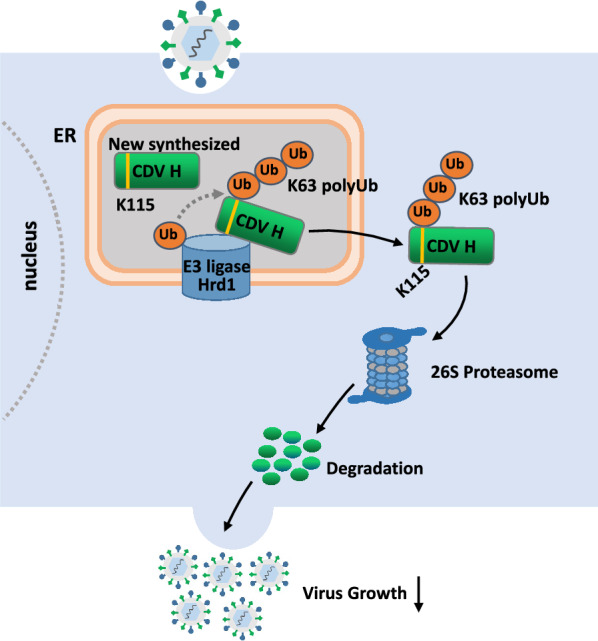
**Proposed model for how Hrd1 influences CDV H protein to inhibit viral replication in cells**. The model shows that the E3 ubiquitin ligase Hrd1 located in ER interacts with CDV H protein, and Hrd1 transfers the activated K63-linked ubiquitin molecules to lysine residues in the 115 site (K115) of substrate protein CDV H. Then, the ubiquitinated CDV H protein is transported to cytoplasm and degraded via the 26 s proteasome. Finally, the degradation of CDV H reduces viral growth.

In summary, more than 89 host proteins associated with the H protein of CDV were screened, and the E3 ubiquitin ligase Hrd1 was identified as an interaction partner of CDV H protein. Hrd1 degraded the CDV H protein and inhibited CDV production, depending on its E3 ligase activity. Furthermore, Hrd1 mediated the ubiquitination of CDV H protein at lysine residue 115 (K115) by a K63-linked ubiquitin chain (Figure 7). A new antiviral strategy may be developed for targeting Hrd1 to prevent and control CDV infection.

## Supplementary Information

**Additional file 1: Information of 412 representative CDV strains of different genotypes used in Additional file 2.**

**Additional file 2: Comparison of K115 in the H protein of 412 strains of different CDV genotypes**. The 412 representative strains of different CDV genotypes were from GenBank, and are listed in Additional file 1. K115 was aligned among the CDV strains of different genotypes by using DNAstar MegAlign software.

**Additional file 3: Effect of Hrd1 on CDV N protein level. 293T cells were transfected with CDV N plasmid alone or together with Myc-Hrd1 for 24 h, and protein levels were determined by Western blot using anti-CDV N, anti-Myc, and anti-GAPDH antibodies**. The level of CDV N protein was quantified by determining band intensities, which were then normalized to the level of GAPDH.
