# Supplementary figures and images for: Host E3 ligase Hrd1 ubiquitinates and degrades H protein of canine distemper virus to inhibit viral replication
Source: Vet Res. 2023 Apr 2;54:30. doi: 10.1186/s13567-023-01163-z (PMC10069049; doi:10.1186/s13567-023-01163-z)

## Slide 1
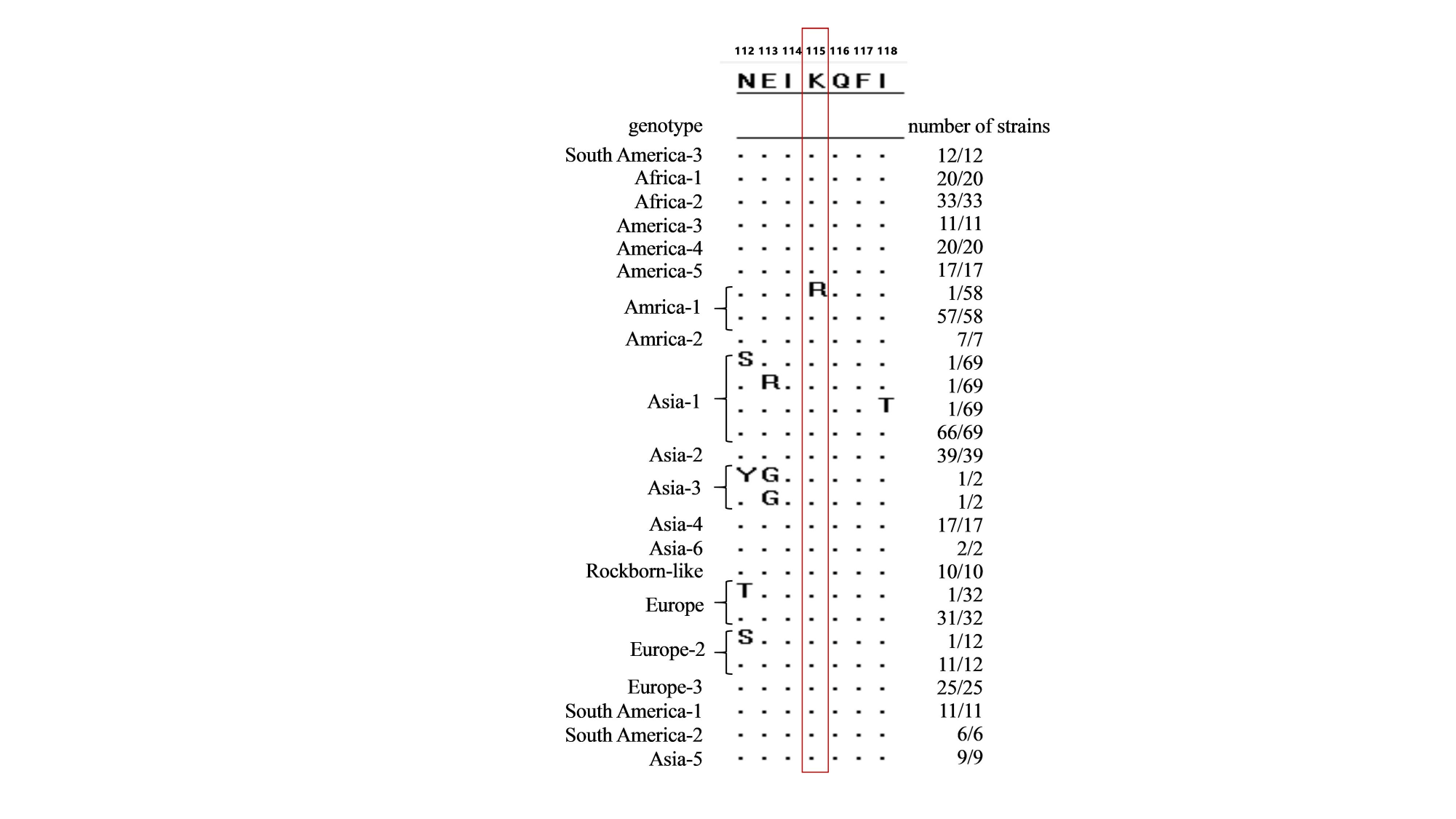

Supplement: Supplementary file 2 — Additional file 2: Comparison of K115 in the H protein of 412 strains of different CDV genotypes. The 412 representative strains of different CDV genotypes were from GenBank, and are listed in Additional file 1. K115 was aligned among the CDV strains of different genotypes by using DNAstar MegAlign software. [file 13567_2023_1163_MOESM2_ESM.pptx]

## Slide 1
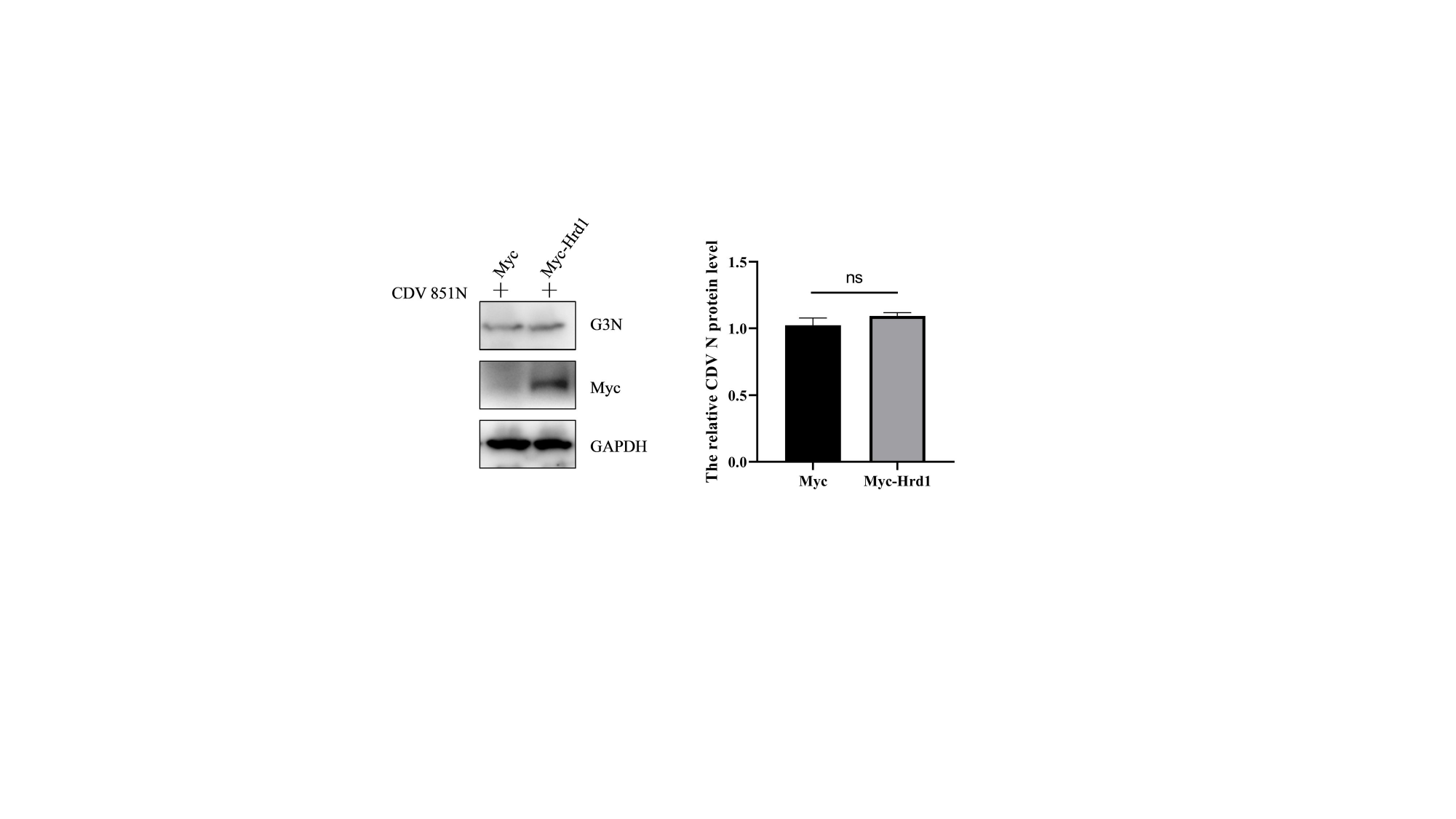

Supplement: Supplementary file 3 — Additional file 3: Effect of Hrd1 on CDV N protein level. 293T cells were transfected with CDV N plasmid alone or together with Myc-Hrd1 for 24 h, and protein levels were determined by Western blot using anti-CDV N, anti-Myc, and anti-GAPDH antibodies. The level of CDV N protein was quantified by determining band intensities, which were then normalized to the level of GAPDH. [file 13567_2023_1163_MOESM3_ESM.pptx]
